# A Visual Assay of a Loop-Mediated Isothermal Amplification Based Vertical Immunoassay for SARS-CoV-2 RNA Detection

**DOI:** 10.3389/fmicb.2022.932698

**Published:** 2022-07-13

**Authors:** Mengtao Yu, Pei Huang, Yuanguo Li, Yumeng Song, Xingqi Liu, Na Feng, Hongli Jin, Yujie Bai, Haili Zhang, Yuanyuan Li, Xianzhu Xia, Yuwei Gao, Hualei Wang

**Affiliations:** ^1^State Key Laboratory for Zoonotic Diseases, Key Laboratory for Zoonosis Research of the Ministry of Education, Institute of Zoonosis, College of Veterinary Medicine, Jilin University, Changchun, China; ^2^Changchun Veterinary Research Institute, Chinese Academy of Agricultural Sciences, Changchun, China

**Keywords:** SARS-CoV-2, reverse transcription loop-mediated isothermal amplification, detection, visualization, variants

## Abstract

SARS-CoV-2 is a novel coronavirus that has caused a global pandemic. To date, 504,907,616 people have been infected and developed coronavirus disease 2019 (COVID-19). A rapid and simple diagnostic method is needed to control this pandemic. In this study, a visual nucleic acid detection method combining reverse transcription loop-mediated isothermal amplification and a vertical flow visualization strip (RT-LAMP-VF) was successfully established and could detect 20 copies/μl of SARS-CoV-2 RNA transcript within 50 min at 61°C. This assay had no cross-reactivity with a variety of coronaviruses, including human coronavirus OC43, 229E, HKU1, NL63, severe acute respiratory syndrome-related coronavirus (SARSr-CoV), Middle East respiratory syndrome coronavirus (MERS-CoV), and bat coronavirus HKU4, exhibiting very high levels of diagnostic sensitivity and specificity. Most strikingly, this method can be used for detecting multiple SARS-CoV-2 variants, including the Wuhan-Hu-1 strain, Delta, and Omicron variants. Compared with the RT-qPCR method recommended by the World Health Organization (WHO), RT-LAMP-VF does not require special equipment and is easy to perform. As a result, it is more suitable for rapid screening of suspected SARS-CoV-2 samples in the field and local laboratories.

## Introduction

In the past 50 years, numerous novel coronaviruses with the ability to infect humans have emerged in succession, causing significant losses and serious threats to human health and even entire public health systems. In particular, with the emergence of severe acute respiratory syndrome coronavirus (SARS-CoV), Middle East respiratory syndrome coronavirus (MERS-CoV), and severe acute respiratory syndrome coronavirus-2 (SARS-CoV-2), coronavirus has attracted the attention of researchers around the world.

SARS-CoV-2 is the seventh coronavirus found to infect humans. Infection with SARS-CoV-2 can cause coronavirus disease 2019 (COVID-19). SARS-CoV-2 has been reported not only in humans but also in dogs, ferrets, cats, tigers, and lions (Gollakner and Capua, 2020; Shi et al., 2020). As of 20 April 2022, SARS-CoV-2 has caused more than 504 million cases of COVID-19 and more than 6.21 million deaths. The World Health Organization (WHO) also classified COVID-19 as a public health emergency of international concern (PHEIC). SARS-CoV-2 is more transmissible than SARS-CoV and MERS-CoV (Chu et al., 2020; Sanche et al., 2020). However, unlike SARS-CoV and MERS-CoV, which were always spread in hospitals, SARS-CoV-2 can also spread rapidly in communities, increasing the difficulty of pandemic prevention and control (Munster et al., 2020). At present, although there are many approved vaccines against SARS-CoV-2, the mutation of the virus reduces their protective effects (Planas et al., 2021). Therefore, the work of pandemic prevention and control must continue.

The laboratory testing strategies for COVID-19 recommended by the WHO include pathogen detection, serological detection (IgG/IgM antibody detection), and nucleic acid detection; among these, nucleic acid detection is the most widely used. The nucleic acid detection for SARS-CoV-2 includes real-time RT-PCR, metagenomics sequencing, and gene editing detection based on CRISPR-Cas12 and CRISPR-Cas13 (Broughton et al., 2020; Corman et al., 2020; Di et al., 2020; Wang et al., 2021), etc. Nucleic acid detection has high sensitivity and accuracy, but there are some shortcomings. For instance, equipment that can perform precise temperature changes is needed, and the assay design methods are complicated. Virus isolation and culture is the internationally recognized gold standard for virological detection. This method has good specificity, but the process is cumbersome and time-consuming. Serological tests, such as enzyme-linked immunosorbent assays (ELISAs) and immunochromatographic strips, have good feasibility, but antibody detection has a certain lag, as antibodies can be detected only after an immune response has been initiated in the host. Therefore, the above methods are not applicable for rapid detection during a pandemic outbreak.

Loop-mediated isothermal amplification (LAMP) is a novel nucleic acid amplification method invented by Notomi et al. (2000) that generally requires two outer primers and two inner primers. This method has high sensitivity and strong specificity. It is particularly attractive due to its convenience and quick operation. The LAMP requires only a portable metal heat block or thermostat water bath to complete the amplification. To date, the RT-LAMP-VF method has been widely used in the detection of various viruses, such as MERS-CoV, Ebola virus, and Rift Valley Fever virus (Oloniniyi et al., 2017; Huang et al., 2018; Han et al., 2020). And this method showed a high sensitivity and specificity (Fu et al., 2011). Conventional LAMP requires electrophoresis to observe the amplification product, and it is easy for false positives to arise because the need to open the lid during operation makes the operation vulnerable to aerosol pollution (Notomi et al., 2000). Some researchers have added magnesium ions to the reaction system, relying on the magnesium ions and pyrophosphate ions in the reaction system to form white magnesium pyrophosphate precipitates, thus achieving visual detection. However, the introduction of magnesium ions reduces the amplification efficiency, and the visual interpretation of magnesium pyrophosphate precipitation is also prone to subjective judgment errors (Mori et al., 2001).

To compensate for the deficiencies of existing methods, we established a nucleic acid visualization detection method based on the N gene of SARS-CoV-2. The SARS-CoV-2 RNA was amplified by RT-LAMP, and the amplification products were detected by a closed vertical flow visualization strip (VF). In addition, two-loop primers were added based on the four primers of the traditional LAMP method to enhance amplification efficiency and specificity (Nagamine et al., 2002). Compared with PCR, the RT-LAMP-VF method does not require precise temperature-changing equipment (Corman et al., 2020) and also has the advantages of simple and rapid operation. Compared with other isothermal amplification technologies, such as nucleic acid sequence amplification, self-sustaining sequence replication, and chain replacement amplification, RT-LAMP-VF is rapid, accurate, and efficient and is suitable for use in local laboratories or at community medical sites (Tomita et al., 2008). This method can provide technical support for the rapid diagnosis of COVID-19.

## Materials and Methods

### Primer Design

**FIGURE 1:**
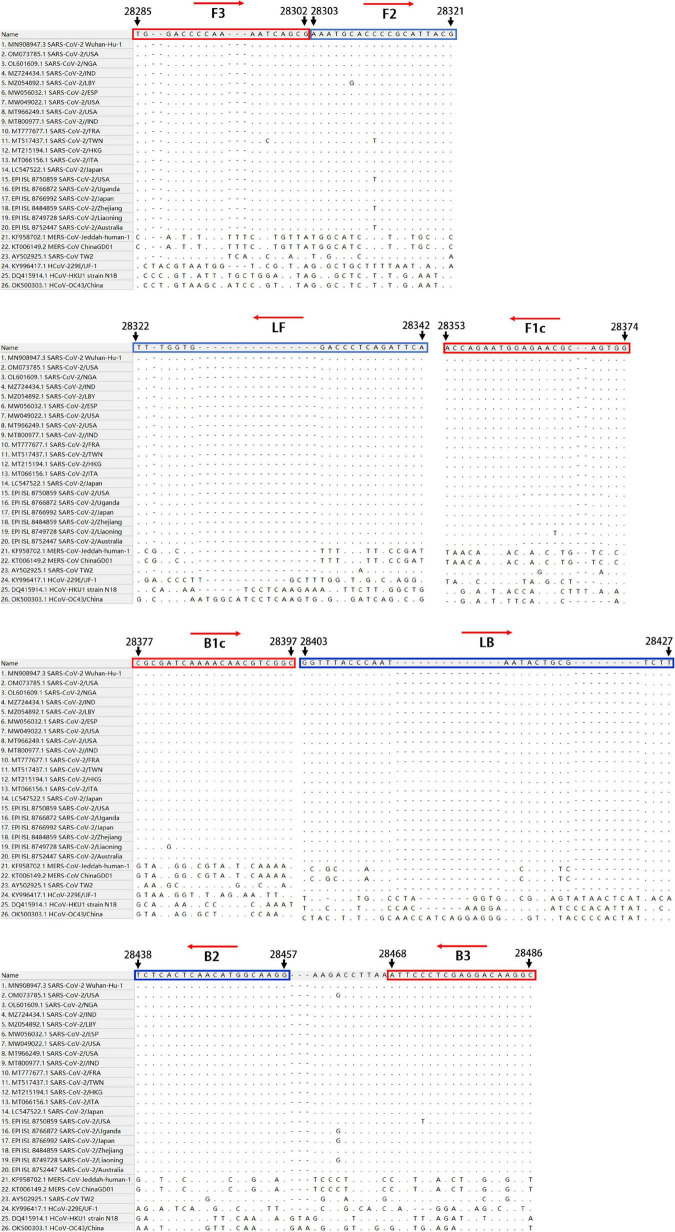
The conserved and specific target were screened in SARS-CoV-2 N gene.

To establish an RT-LAMP-VF method for the detection of SARS-CoV-2, we aligned the whole-genome sequences of 20 SARS-CoV-2 pandemic strains, including the Wuhan-Hu-1 strain, Delta, and Omicron variants, published in the GenBank and GISAID databases from 2019 to 2022 with MEGALIGN 8.0. The alignment showed that the N gene was highly conserved. Therefore, the N gene sequence of SARS-CoV-2 was compared with those of the highly homologous MERS-CoV, SARS-CoV, and SARSr-CoV viruses, and conserved gene fragments were selected as targets. Six primers suitable for SARS-CoV-2 RT-LAMP were designed by using Primer Explorer V5^1^ with the conserved region of the N gene as the template (Figure 1). The RT-LAMP-VF assay requires three sets of primers, including two outer primers (F3 and B3), two inner primers (FIP and BIP), and two loop primers (LF and LB). The outer and inner primers are conventional primers without labels, and fluorescein isothiocyanate (FITC) and biotin were labeled at the 5′ ends of LF and LB, respectively (Table 1). All primers were synthesized by Bao Biological, Co., Ltd. (Dalian, China).

**TABLE 1:** Primer and probe sequences for the SARS-CoV-2 RT-qPCR and RT-LAMP assays.

Method	Genomic target	Primer or probe	Primer Position	Sequence (5′-3′)
RT-LAMP	N	F3	28285–28302	TGGACCCCAAAATCAGCG
		FIP(F1c + F2)	28353–28374	CCACTGCGTTCTCCATTCTGGT
			28303–28321	AAATGCACCCCGCATTACG
		B3	28468–28486	GCCTTGTCCTCGAGGGAAT
		BIP(B1c + B2)	28377–28397	CGCGATCAAAACAACGTCGGC
			28438–28457	CCTTGCCATGTTGAGTGAGA
		LF	28322–28342	FITC-TGAATCTGAGGGTCCACCAAA
		LB	28403–28427	Biotin-GGTTTACCCAATAATACTGCGTCTT
RT-qPCR	N	Forward primer	28881–28902	GGGGAACTTCTCCTGCTAGAAT
		Reverse primer	28958–28979	CAGACATTTTGCTCTCAAGCTG
		Probe	28934–28953	5′-FAM-TTGCTGCTGCTTGACAGATT-TAMRA-3′

### Cloning of Recombinant Plasmid and Viral RNA

The recombinant plasmid pUC57-N containing the SARS-CoV-2 N gene (GenBank number: MN908947.3) was synthesized by Sangon Biotech Co., Ltd. (Shanghai, China) and transformed into *E. coli* DH5α competent cells, which were then cultured at 37°C. The recombinant plasmid was purified using a plasmid rapid extraction kit (TIANGEN Company, Beijing, China) and stored at −20°C. The concentration of the purified pUC57-N was 297 ng/μl.

RNA transcripts of the N gene (from 28274 to 29533) of SARS-CoV-2 Wuhan-Hu-1 (GenBank No. MN908947.3), Delta variant (EPI_ISL_8038262), and Omicron (EPI_ISL_8752447) were synthesized by Sangon Biotech Co., Ltd. (Shanghai, China). Total RNA of SARS-related coronaviruses (SARSr-CoV JTMC15 strain) and HKU4 was extracted from two intestinal tissue samples of bats infected with the different corresponding viruses and then stored in our laboratory. The RNA of respiratory secretions from BALB/c mice and cynomolgus monkeys infected with SARS-CoV-2 is stored at Changchun Veterinary Research Institute. Total RNA of MERS-CoV strain GD01 was stored in our laboratory.

The total nucleic acids of multiple respiratory pathogens, such as human coronavirus OC43, 229E, NL63, and HKU1 (Table 2), were purified from the NATtrol RP Multimarker Controls kit (ZeptoMetrix Corporation, Franklin, United States) by the TIANamp Virus DNA/RNA Kit (TIANGEN Company, Beijing, China).

**TABLE 2:** Respiratory pathogens included in the NATtrol RP multimarker controls kit.

RP1 Respiratory virus	Strain	RP2 Respiratory virus	Strain
Influenza A H3N2	Brisbane/10/07	Influenza A H1	New Caledonia/20/99
Influenza A H1N1	NY/02/2009	Influenza B	Florida/02/06
Rhinovirus	Type 1A	RSV	Type A
Adenovirus	Type 3	Parainfluenza	Type 2
Parainfluenza	Type 1	Parainfluenza	Type 3
Parainfluenza	Type 4	Coronavirus	HKU1 (recombinant)
Metapneumovirus	Peru 6–2003	Coronavirus	OC43
C. pneumoniae	CWL-029	Coronavirus	NL63
M. pneumoniae	M129	Coronavirus	229E
Coxsackievirus	Type A1	Bordetella pertussis	A639

### Establishment and Optimization of the RT-LAMP-VF Reaction System

Different concentrations of the recombinant plasmid pUC57-N were used as templates to estimate the RT-LAMP-VF method. The reaction solution with a total volume of 25 μl was configured and the main containing primer, AMV reverse transcriptase (Promega, Beijing, China), Bst2.0 WarmStart^®^ DNA polymerase (New England Biolabs, Beijing, China), and the template was prepared, moreover, the other components and their information are presented in Table 3. The reaction mixture was mixed and amplified at 61°C for 50 min. All amplification products were detected with a disposable nucleic acid visualization detection device (Ustar Biotech, Co., Ltd., Hangzhou, China).

**TABLE 3:** Reaction system of RT-LAMP-VF assay.

Composition	Final concentration	Manufacturer
dNTP	1.4 mM	Bao Biological, Dalian, China
MgSO4	4 mM	Sigma, Shanghai, China
10 × Buffer	2.5 μL	New England Biolabs, Beijing, China
Betaine	0.2 M	Sigma, Shanghai, China
Bst2.0 WarmStart DNA polymerase	8 U	New England Biolabs, Beijing, China
AMV reverse transcriptase	5 U	Promega, Beijing, China
FIP/BIP	0.2 μM	Bao Biological, Dalian, China
F3/B3	0.05 μM	Bao Biological, Dalian, China
LF/LB	0.1 μM	Bao Biological, Dalian, China
Template	5 μL	Sangon Biotech, Shanghai, China
DEPC-treated water	7.25 μl	Solarbio, Beijing, China

The recombinant plasmid pUC57-N was used as the amplification template, and five different amplification temperatures (59, 61, 63, 65, and 67°C) were tested to determine the optimal amplification temperature. The amplification was performed at constant temperature for 50 min, and the reaction results were analyzed. After determining the optimal amplification temperature, four different amplification times (30, 40, 50, and 60 min) were tested at the optimal temperature. Each amplification reaction was repeated three times.

SARS-CoV-2 RNA was used as the RT-LAMP-VF amplification template to verify the above-optimized conditions, and samples with different RNA copy numbers were detected under the optimal conditions. Each amplification reaction was repeated three times.

### RT-LAMP-VF Assay Specificity and Sensitivity Evaluation

To evaluate the specificity of the RT-LAMP-VF method, we extracted SARSr-CoV and HKU4 RNA from the intestinal tissues of two bats with an RNA extraction kit and extracted the RNAs of various respiratory pathogens from the RP1 and RP2 kits of NATtrolTM RP Multimarker control. Then, the above nucleic acids were tested to evaluate the specificity of the RT-LAMP-VF.

The RNA transcript samples were diluted to 2 × 10^6^, 10^5^, 10^4^, 10^3^, 10^2^, 10^1^, 10^0^, and 10^–1^ copies/μl by 10-fold serial dilution. The sensitivity of the RT-LAMP-VF method was evaluated by assaying samples with different RNA copy numbers to obtain a lower detection limit.

The synthetic RNA transcripts of SARS-CoV-2 mutant strains, including Wuhan-Hu-1 strain, Delta, and Omicron variants, were used to evaluate the RT-LAMP-VF assay, and the RNA concentration of each mutant strain was 20 copies/μl.

### The Gold Standard for Detection of SARS-CoV-2 RNA

According to a protocol of the gold standard for SARS-CoV-2 recommended by the Chinese center for Disease Control and Prevention, the RT-qPCR was performed. The sequence of primers and probes was described in Table 1. Reactions were conducted in a 25 μl volume following the instructions of TaqMan™ qPCR Master Mix (Applied Biosystems, Foster, CA, United States). The reaction cycle parameters were set as follows: reverse transcription at 50°C for 10 min, denaturation at 95°C for 5 min, and then 40 amplification cycles of 95°C for 10 s and 55°C for 40 s. After completion of amplification, if the cycle threshold (CT) value is lower than 37, the results are judged to be positive. The result is negative when the CT value is greater than 40. When the CT value is between 37 and 40, it is suggested to be detected again. Each group included one no-template control.

### Detection of Clinical Samples by the RT-LAMP-VF Method

The RNA of respiratory secretions from BALB/c mice and cynomolgus monkeys infected with SARS-CoV-2 was collected, including 6 respiratory secretions from BALB/c mice, 5 throat swab samples from cynomolgus monkeys, and 20 respiratory secretions from healthy BALB/c mice and cynomolgus monkeys. The RNA of the above samples was detected by RT-LAMP-VF and RT-qPCR. Each amplification reaction was repeated three times.

## Results

### RT-LAMP-VF Assay Development

**FIGURE 2:**
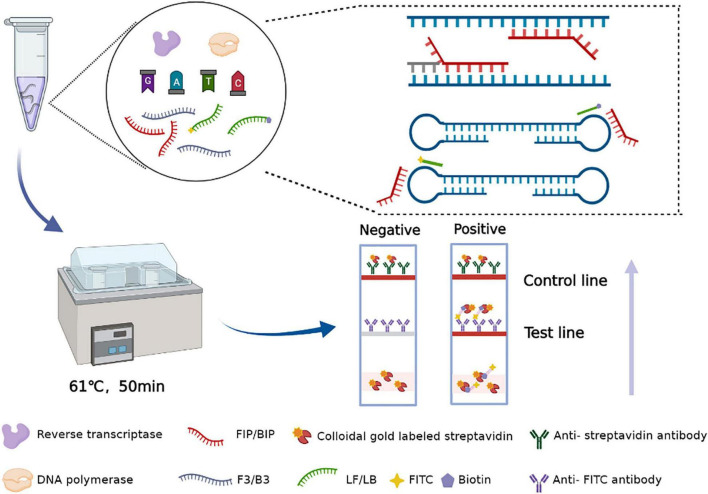
Schematic illustration of the RT-LAMP-VF assay targeting the SARS-CoV-2 N gene.

In this assay, the amplification product is placed into a disposable nucleic acid detection device, and the detection device is closed. The closed vertical flow nucleic acid detection device consists of a test strip and diluent. In this device, the control line and detection line are labeled with anti-streptavidin antibody and anti-FITC antibody, respectively. Simultaneously, gold particles are incubated on the binding pad, which was coated with streptavidin. After the tube containing the amplification products is placed into the device, the amplicons that are labeled with biotin can bind to the colloidal gold particles conjugated with streptavidin to form a complex. Then, the complex labeled with FITC is captured by an anti-FITC antibody on the test line of the strip, and the test results are produced. Abundant gold particles gather to form visible lines. The results are observable by the naked eye within 5 min, without additional dyes or fluorescence signal acquisition equipment. When both the detection line and the control line show red bands, the results are judged to be positive; if only the control line appears as a red band, the result is negative; if no red band appears at the control line, the test result is considered invalid (Figure 2).

### Optimizing the RT-LAMP-VF Reaction Conditions

The reaction conditions of the RT-LAMP-VF method were optimized with different concentrations of recombinant plasmid pUC57-N as the amplification template.

The RT-LAMP amplification reaction solution was tested at five different amplification temperatures (59, 61, 63, 65, and 67°C) with amplification at constant temperature for 50 min. According to the reaction results, the sensitivity of the RT-LAMP reaction was optimal at 65°C, at which temperature the recombinant plasmid pUC57-N could be detected at a minimum of 2 × 10^1^ copies/μl (Table 4).

**TABLE 4:** Reaction temperature optimization for RT-LAMP-VF.

Temperature/°C	Recombinant plasmids dilution (2 × copies/μl)								
	10^7^	10^6^	105	10^4^	10^3^	10^2^	10^1^	10^0^	N
59	+	+	−	−	−	−	−	−	−
61	+	+	+	+	+	+	−	−	−
63	+	+	+	+	+	+	−	−	−
65	+	+	+	+	+	+	+	−	−
67	+	+	+	+	+	+	−	−	−

*Three replications were performed for each trial.*

To determine the optimal amplification time, 25 μl of the amplification reaction solution was amplified at 65°C for 30, 40, 50, and 60 min. The lowest concentration of recombinant plasmid pUC57-N, 2 × 10^1^ copies/μl, could be detected when the amplification time was 50 min at 65°C. Therefore, 50 min was considered the optimal amplification time for the RT-LAMP reaction (Table 5).

**TABLE 5:** Reaction time optimization for the RT-LAMP-VF assay.

Time/min	Recombinant plasmids dilution (2 × copies/μl)								
	10^7^	10^6^	10^5^	10^4^	10^3^	10^2^	10^1^	10^0^	N
30	+	+	+	+	+	−	−	−	−
40	+	+	+	+	+	+	−	−	−
50	+	+	+	+	+	+	+	−	−
60	+	+	+	+	+	+	+	−	−

*Three replications were performed for each trial.*

Since SARS-CoV-2 is an RNA virus, RNA transcripts were used as amplification templates to verify that the optimized conditions were suitable for RT-LAMP-VF detection of SARS-CoV-2. At least 2 × 10^1^ copies/μl of RNA transcripts were detected after amplification at 61°C for 50 min. Therefore, 61°C was considered the optimal amplification temperature (Table 6).

**TABLE 6:** Reaction temperature optimization of RNA for RT-LAMP-VF assay.

Temperature/°C	Recombinant plasmids dilution (2 × copies/μl)								
	10^7^	10^6^	10^5^	10^4^	10^3^	10^2^	10^1^	10^0^	N
59	+	+	−	−	−	−	−	−	−
61	+	+	+	+	+	+	+	−	−
63	+	+	+	+	+	+	−	−	−
65	+	+	+	+	+	+	−	−	−

*Three replications were performed for each trial.*

### Specificity and Sensitivity of the RT-LAMP-VF Assay

**FIGURE 3:**
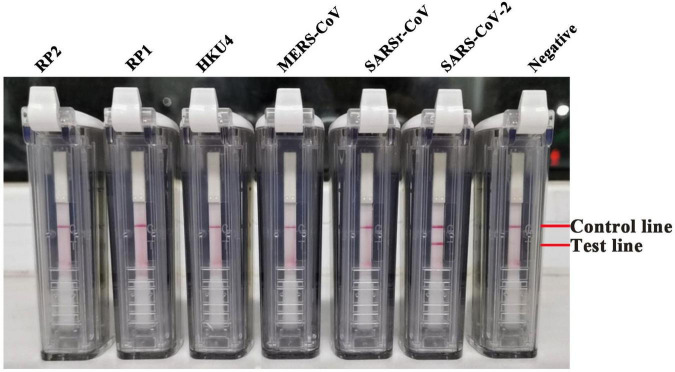
The specificity of the RT-LAMP-VF assay has been examined through the respiratory pathogens. Nucleic acids from a variety of human coronaviruses and other respiratory pathogens were detected by the RT-LAMP-VF assay.

The specificity of the RT-LAMP-VF method was evaluated by testing RNA samples of MERS-CoV, SARSr-CoV, HKU4, and various respiratory pathogens and the RNA transcripts of SARS-CoV-2 as templates. The results showed that only the RNA transcript of SARS-CoV-2 produced a positive result, and the RT-LAMP-VF assay had no cross-reaction with SARSr-CoV, HKU4, HKU1, OC43, 229E, or others (Figure 3).

**FIGURE 4:**
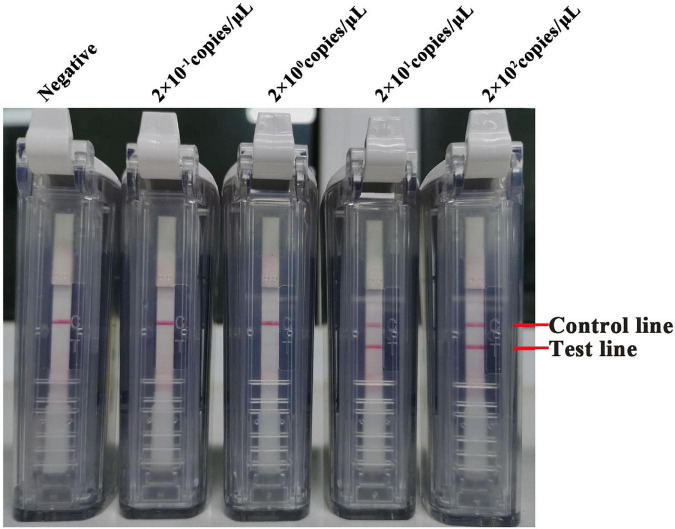
The sensibility of the RT-LAMP-VF assay targeting the N gene. The limit of detection of the RT-LAMP-VF assay using 10-fold serially of SARS-CoV-2 RNA transcripts.

**FIGURE 5:**
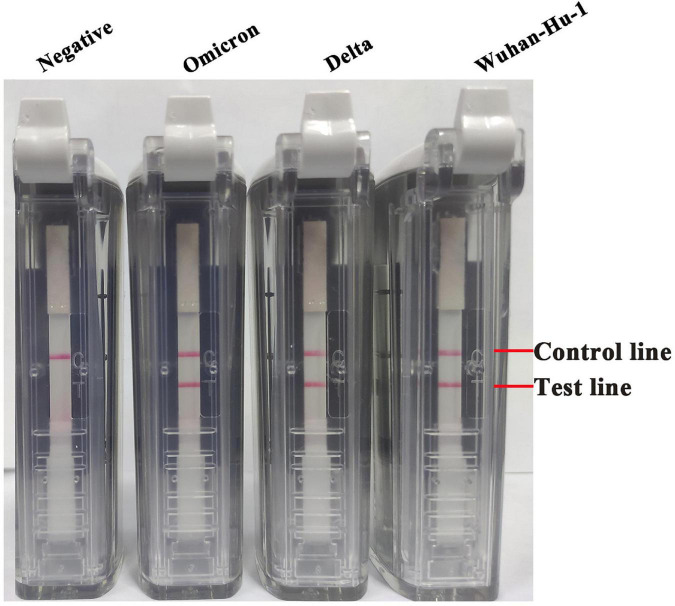
The applicability of the RT-LAMP-VF assay detected multiple SARS-CoV-2 mutants. Multiple RNA transcripts of variants including Wuhan-Hu-1 strain, Delta, and Omicron were used to evaluate the RT-LAMP-VF assay, and the concentration of each RNA transcript was 20 copies/μl.

Tenfold serial dilutions of the synthesized RNA transcripts (ranging from 2 × 10^6^ to 2 × 10^–1^ copies/μl) were subjected to the RT-LAMP-VF assay to assess its detection limit. Three replicates were performed for each trial. The amplification was performed at 61°C for 50 min, and the method can detect SARS-CoV-2 RNA transcripts at as few as 20 copies/μl (Figure 4). For evaluating the applicability of the RT-LAMP-VF assay, multiple variants were used for evaluating this method, including the Wuhan-Hu-1 strain and the Delta and Omicron variants. As shown in Figure 5, RT-LAMP-VF can be used to detect Delta and Omicron variants, and the detection limit for all was 20 copies/μl RNA transcripts. This proves that the RT-LAMP-VF assay has good applicability in the detection of Delta and Omicron mutants.

### Evaluation of the RT-LAMP-VF Method Using Clinical Samples

The RNA of respiratory secretions from BALB/c mice and cynomolgus monkeys infected with SARS-CoV-2 and from 20 healthy BALB/c mice and cynomolgus monkeys was also studied. Using the above method, all 11 positive samples were accurately detected. The results show that RT-LAMP-VF can be applied to the detection of clinical samples. The RT-LAMP-VF assay had a sensitivity of 100% (95% CI, 0.678–1.00) and a specificity of 100% (95% CI, 0.88–1.00). The coincidence rate between the RT-qPCR and RT-LAMP-VF assays was 100%, indicating that the assays showed high consistency (Table 7).

**TABLE 7:** The sensitivity and specificity of the RT-LAMP-VF assay were evaluated in viral RNA specimens.

The RT-LAMP-VF assay panel	Two gold standard real time RT-PCR assays		Sensitivity	Specificity	Concordance rate (%)
			
	Positive samples (*N* = 11)	Negative samples (*N* = 20)	Concordance rate (95% CI)		
Positive	11	0	100% (67.8–100%)	100% (88–100%)	100%
Negative	0	20			

## Discussion

SARS-CoV-2 first emerged in December 2019, and it has since been spreading rapidly worldwide, causing the ongoing global COVID-19 pandemic. In some areas with large populations, once disease spread begins, there is a demand for immediate nucleic acid testing at various sites, even multiple rounds of full nucleic acid testing, and there is insufficient testing personnel and equipment for the large amount of testing required. In addition, some localities have set up temporary sampling collection sites for nucleic acid detection, following which the collected samples are sent to a central laboratory for screening. To ensure the quality of samples, reduce the risk of personal infection caused by sample transportation, and meet the need for rapid on-site diagnosis of COVID-19, we established a rapid and simple RT-LAMP-VF detection method.

In this article, we established and characterized a RT-LAMP-VF detection method for the SARS-CoV-2 N gene. This assay takes only 50 min to detect an RNA transcript at 2 × 10^1^ copies/μl. Compared with other SARS-CoV-2 molecular methods, such as PCR and RT-qPCR, the RT-LAMP-VF method is fast and can be performed at a constant temperature. Yan and colleagues developed an RT-LAMP method for detecting SARS-CoV-2 that targeted the ORF1ab gene and S gene (Yan et al., 2020). The amplification results were interpreted with a real-time turbidity meter or visually. The primers targeting the ORF1ab gene and S gene could detect 2 × 10^1^ copies/μl and 2 × 10^2^ copies/μl SARS-CoV-2 RNA, respectively. Huang and colleagues also developed RT-LAMP methods for SARS-CoV-2 targeting the ORF1ab, N, and S genes (Huang et al., 2020). The amplification results were generated by a colorimetric method based on the pH indicator phenol red. The minimum detection level was 2 copies of SARS-CoV-2 RNA, but the assessment of the colorimetric results was not intuitive when the sample was weakly positive, and the results were easily affected by the pH of the buffer.

Unlike the RT-LAMP methods mentioned above, the RT-LAMP-VF method introduces additional loop primers into the amplification system, which improves the overall amplification efficiency. Furthermore, the method used FITC and biotin to label the 5’ ends of loop primers LF and LB, which allowed the amplification results to be visualized on a closed vertical flow nucleic acid detection device. Conventional LAMP makes the operation vulnerable to aerosol pollution (Notomi et al., 2000). To make up for these deficiencies, we used the closed vertical flow nucleic acid detection device, it not only showed the RT-LAMP product but also avoided the problem of aerosol contamination of the LAMP reaction. However, the RT-LAMP-VF method also has some shortcomings. The container matched with the disposable nucleic acid detection device is a single PCR tube, so the detection amount of a single sample is limited, and high-throughput detection of a large number of samples cannot be realized.

With the continuation of the COVID-19 epidemic, SARS-CoV-2, similar to other RNA viruses, continues to mutate, and new variants continue to appear all over the world, including some variants with stronger infectivity and transmission potential, which further increases the difficulty of epidemic prevention and control. D614G was the earliest identified mutation of SARS-CoV-2, affecting the gene encoding the S protein (Corum and Zimmer, 2021). A variant named 01, which originated from N501y, was found in the United Kingdom in December 2020 and eventually called SARS-CoV-2 VOC 202012Accord 01 or B.1.1.7. Subsequently, the mutant B.1.351 was also found to have a variety of mutations (Madhi et al., 2021). Compared with the original SARS-CoV-2, many nucleotides have been replaced in these variants, leading to amino acid mutations, and most of these mutations were located in the S protein (Faria et al., 2021; Leung et al., 2021; Rambaut et al., 2021). The loss of certain sites in the S protein or nucleotide sequence can affect the performance of PCR detection and diagnostic methods that rely on the S gene as the target (Corum and Zimmer, 2021). Our RT-LAMP-VF detection method targeted a specific fragment of the N gene of SARS-CoV-2 and can be used for detecting many SARS-CoV-2 variants, including the original strain, the Delta variant, and the Omicron variant. To test whether the mutation of the virus strain affected the accuracy of RT-LAMP-VF detection, we tested our method on SARS-CoV-2 mutant RNA transcripts. The results showed that the sensitivity of this method was not significantly different for the Delta and Omicron mutants.

Since the outbreak of the pandemic in late 2019, multifarious detection kits have emerged to effectively control the spread of the pandemic, but the targets of different methods are different. The researchers analyzed and compared the sensitivity and detection efficiency of RT-qPCR primers and probes for SARS-CoV-2 (Vogels et al., 2020). Among them, the primer sensitivity and detection efficiency for the N gene and ORF1a/b gene were the highest. A previous study showed that RT-LAMP for the detection of the SARS-CoV-2 N gene could specifically detect SARS-CoV-2 RNA and did not cross-react with related coronaviruses (Baek et al., 2020). In this paper, we also analyzed the RT-LAMP-VF detection method for the N gene of SARS-CoV-2. The specificity of the method was evaluated by application to samples of other coronaviruses, including SARSr-CoV, MERS-CoV, HKU4, HKU1, OC43, 229E, and NL63. Our results were consistent with the gold standard of RT-qPCR.

Since COVID-19 has the characteristics of rapid propagation, wide distribution, and repeated outbreaks, it is of great significance to develop a rapid and simple method to improve the control of COVID-19. In this paper, the RT-LAMP-VF method for the N gene of SARS-CoV-2 takes only 50 min and can detect RNA transcript at 20 copies/μl. The RT-LAMP-VF method established in this paper is suitable for the rapid detection of new or recurrent infectious diseases and can be considered the best alternative to RT-qPCR (Keikha, 2018). In summary, this method has broad applicability and is expected to achieve on-site real-time detection without the need to transport samples, making it especially useful for screening in airports and train stations.

## Data Availability Statement

The original contributions presented in this study are included in the article, further inquiries can be directed to the corresponding authors.

## Author Contributions

HW and PH designed the experiments. MY, PH, and HW wrote the manuscript. MY, YS, and XL performed the experiment. HJ and YB analyzed the data. HZ, YYL, YG, and HW reviewed the manuscript. YGL gave help in the field of RNA extraction. All authors have read and agreed to the published version of the manuscript.

## Conflict of Interest

The authors declare that the research was conducted in the absence of any commercial or financial relationships that could be construed as a potential conflict of interest.

## Publisher’s Note

All claims expressed in this article are solely those of the authors and do not necessarily represent those of their affiliated organizations, or those of the publisher, the editors and the reviewers. Any product that may be evaluated in this article, or claim that may be made by its manufacturer, is not guaranteed or endorsed by the publisher.

## Footnotes

1
http://primerexplorer.jp/lampv5e/index.html
